# Myocardial Approximate Spin-lock Dispersion Mapping using a Simultaneous *T*_2_ and *T*_*RAFF*2_ Mapping at 3T MRI

**DOI:** 10.1109/EMBC48229.2022.9871465

**Published:** 2022-07

**Authors:** Joao Tourais, Omer Burak Demirel, Qian Tao, Iain Pierce, George D Thornton, Thomas A Treibel, Mehmet Akcakaya, Sebastian Weingärtner

**Affiliations:** 1 Department of Imaging Physics, Delft University of Technology (TU Delft), Delft, Netherlands; 2 Department for Electrical and Computer Engineering, Minneapolis, MN, USA; 3 Center for Magnetic Resonance Research, University of Minnesota, Minneapolis, MN, USA; 4 Barts Heart Centre and Institute for Cardiovascular Science, University College London, London, United Kingdom; 5 HollandPTC consortium – Erasmus Medical Center, Rotterdam, Holland Proton Therapy Centre, Delft, Leiden University Medical Center (LUMC), Leiden and Delft University of Technology, Delft, The Netherlands

## Abstract

Ischemic heart disease (IHD) is one of the leading causes of death worldwide. Myocardial infarction (MI) represents a third of all IHD cases, and cardiac magnetic resonance imaging (MRI) is often used to assess its damage to myocardial viability. Late gadolinium enhancement (LGE) is the current gold standard, but the use of gadolinium-based agents limits the clinical applicability in some patients. Spin-lock (SL) dispersion has recently been proposed as a promising non-contrast biomarker for the assessment of MI. However, at 3T, the required range of SL preparations acquired at different amplitudes suffers from specific absorption rate (SAR) limitations and off-resonance artifacts. Relaxation Along a Fictitious Field (RAFF) is an alternative to SL preparations with lower SAR requirements, while still sampling relaxation in the rotating frame. In this study, a single breath-hold simultaneous *T*_*RAFF*2_ and *T*_2_ mapping sequence is proposed for SL dispersion mapping at 3T. Excellent reproducibility (coefficient of variations lower than 10%) was achieved in phantom experiments, indicating good intrascan repeatability. The average myocardial *T*_*RAFF*2_, *T*_2_, and SL dispersion obtained with the proposed sequence (68.0±10.7 ms, 44.0±4.0 ms, and 0.4±0.2 × 10^*−*4^ *s*^2^, respectively) were comparable to the reference methods (62.7±11.7 ms, 41.2±2.4 ms, and 0.3±0.2 × 10^*−*4^ *s*^2^, respectively). High visual map quality, free of *B*_0_ and $B_{1}^{+}$ related artifacts, for *T*_2_, *T*_*RAFF*2_, and SL dispersion maps were obtained in phantoms and in vivo, suggesting promise in clinical use at 3T.

## I. INTRODUCTION

Magnetic resonance imaging (MRI) established itself as an important modality in the clinical and preclinical evaluation of ischemic heart disease (IHD) [1]. Currently, the gold standard for the detection of chronic or irreversible injury of the myocardium is late gadolinium enhancement (LGE) MRI. However, LGE requires the use of gadolinium-based contrast agents (GBCA). GBCAs pose the risk of deposition in the brain and nephrogenic systemic fibrosis (NSF), and are, therefore, contraindicated in patients with renal dysfunction [2]. With the emergence of quantitative cardiac MR methods, quantitative myocardial tissue characterization has been proposed as a non-contrast alternative for scar assessment. For example, *T*_2_ relaxation time mapping has been implemented to detect edema in acute myocardial infarction (MI) [3]. However, detection of fibrosis and permanent tissue damage has so far remained out of reach for conventional relaxometry. Recently, rotating frame relaxometry, e.g. *T*_1*ρ*_ mapping [4], has revealed new insights about the macro-molecular content of biological tissues and has been used to quantify myocardial fibrosis without the injection of contrast agents. However, similar to conventional relaxometry, *T*_1*ρ*_ is not specific and is affected by numerous underlying physical effects.

Spin-lock (SL) dispersion has recently been proposed as a new imaging biomarker for the detection of MI [5]. SL dispersion has been shown to isolate chemical exchange contributions to SL relaxation, potentially presenting a more specific imaging biomarker [6]. SL dispersion is usually obtained by acquiring a range of *T*_1*ρ*_ maps at different amplitudes (frequencies). However, at 3T, the specific absorption rate (SAR) limits the use of high-frequency SL pulses, and long SL preparations suffer from strong off-resonance-induced artifacts. Adiabatic *T*_1*ρ*_ may be used to avoid off-resonance effects, but its use in dispersion mapping is hampered because variable effective field strength and sweep duration complicate the relation to a well-defined SL frequency. Relaxation Along a Fictitious Field (RAFF) is an alternative to SL pulses with lower SAR requirements [7]. RAFF operates in a sub-adiabatic regime with constant effective field strength and an identical, constant fictitious field strength leading to uniform sweeps. In previous studies, RAFF has been shown to preserve excellent sensitivity to acute MI and chronic scar [8], but its use for dispersion mapping remains unstudied.

In this work, we sought to provide a method for the assessment of myocardial SL dispersion at 3T using RAFF in a single breath-hold (BH). The proposed technique is evaluated in phantom measurements and initial in vivo images of a healthy subject.

## II. METHODS

### A. Sequence Design

**Fig. 1.**
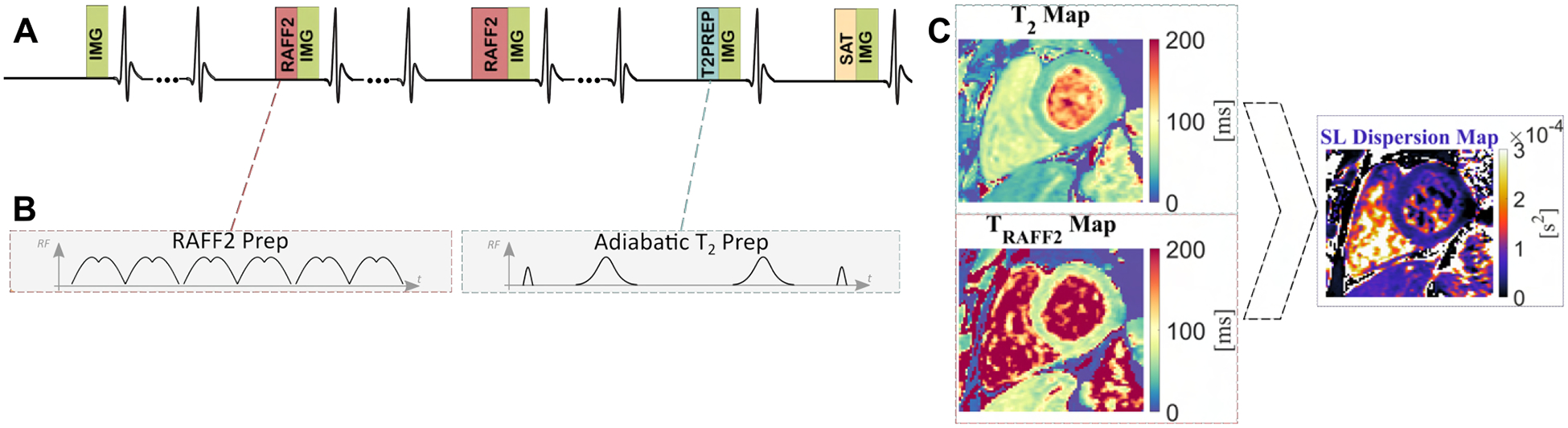
A) Pulse sequence diagram for the proposed simultaneous myocardial *T*_*RAFF*2_ and *T*_2_ mapping sequence. B) Schematic representation of the amplitude of the RF pulses. C) The difference between the *T*_*RAFF*2_ and *T*_2_ map divided by the RAFF2 pulse amplitude results in an approximation for a spin-lock (SL) dispersion map.

In the proposed simultaneous *T*_*RAFF*2_ and *T*_2_ mapping sequence, five balanced steady-state free precession (bSSFP) images with different magnetization preparations were acquired during a single 16s BH (Fig. 1).

A first image acquired without preparation is followed by images prepared with two RAFF2 preparation blocks [7] (*T*_*RAFF*2*p*_) with different durations and one *T*_2_ preparation (*T*_2*p*_) duration, each preceded by a 4 s rest period. A saturation-prepared image was acquired in the subsequent heartbeat to capture the effect of imaging pulses on the magnetization curve. Simultaneous *T*_*RAFF*2_ and *T*_2_ maps were obtained using a four-parameter fit:

(1) $$S\left(T_{\text{RAFF}2p},T_{2p},A,B\right)=\{\begin{array}{l} A\cdot e^{-\frac{T_{\text{RAFF}2p}}{T_{\text{RAFF}2}}}+B\\ A\cdot e^{-\frac{T_{2p}}{T_{2}}}+B \end{array}$$

SL dispersion maps were calculated by the difference between *T*_2_ (zero-frequency SL) and *T*_*RAFF*2_ (approximation for SL relaxation) times normalized by the RAFF frequency as the assumed SL frequency.

All imaging was performed on a 3T MRI scanner (Prisma, Siemens Healthineers, Erlangen, Germany) with a 28-channel receiver coil array. This study was approved by the local institutional review board and written informed consent was obtained before each examination.

The imaging parameters were kept constant for phantom and in vivo measurements: FOV: 320 × 255 *mm*^2^; In-plane resolution: 1.7 × 1.7 *mm*^2^; Slice thickness: 8 mm; GRAPPA factor/Reference lines: 2/24; TE/TR: 1.6/3.2 ms; Flip Angle: 70°; Readout type: bSSFP. SAR burden remained under the threshold for standard operation and no first-level mode was enabled.

### B. Phantom experiments

Phantom measurements were performed on the T1MES phantom [9] to evaluate the accuracy and precision of the proposed sequence against regular *T*_*RAFF*2_ and *T*_2_ mapping. Simultaneous *T*_*RAFF*2_ and *T*_2_ maps were obtained using the proposed sequence with *T*_*RAFF*2*p*_ times of 12.9 and 25.7 ms (RF pulse power of 625 Hz) and *T*_2*p*_ of 50 ms. The SL dispersion map obtained with the proposed sequence was achieved following Eq. 1.

A reference *T*_*RAFF*2_ map was obtained using *T*_*RAFF*2*p*_ = 0, 12.9, 25.7, and 38.6 ms and an image preceded by a saturation pulse. Reference *T*_2_ map was acquired with *T*_2*p*_ of 0, 50, 50, 50 ms, followed by an image preceded by a saturation pulse [10]. Reference SL dispersion map was calculated using the individual reference *T*_2_ and *T*_*RAFF*2_ maps. The imaging parameters were kept identical to those of the proposed method.

Correlation and Bland-Altman plots were obtained to compare the simultaneous and the individual *T*_*RAFF*2_, *T*_2_, and SL dispersion values.

To assess intrascan reproducibility, three scan repetitions were performed in separate acquisitions in the phantom experiments, and the coefficient of variation (CV) across all measurements was computed.

### C. In vivo experiments

In vivo *T*_*RAFF*2_ and *T*_2_ maps were obtained using the proposed sequence for a healthy subject (male, 35 years) with a BH of 16 s. The *T*_*RAFF*2*p*_ and *T*_2*p*_ preparation times were identical to the sequence used in the phantom experiments. All baseline in vivo images were registered using groupwise registration [11] to minimize motion within and across the separate BH and the SL dispersion map was computed (Eq. 1). Standard deviation (SD) maps were obtained from the fit residuals to obtain a spatially resolved estimation of the precision of the proposed sequence [12]. In two separate 16 BH acquisitions, individual *T*_*RAFF*2_ and *T*_2_ reference maps were obtained using the same sequence parameters as in the phantom experiments. Reference SL dispersion map was obtained from the individual single-parameter reference maps. Visual image quality and mean (± std) myocardial quantitative values were compared between the proposed sequence and the individual reference maps for *T*_*RAFF*2_, *T*_2_, and SL dispersion.

## III. RESULTS

### A. Phantom

**Fig. 2.**
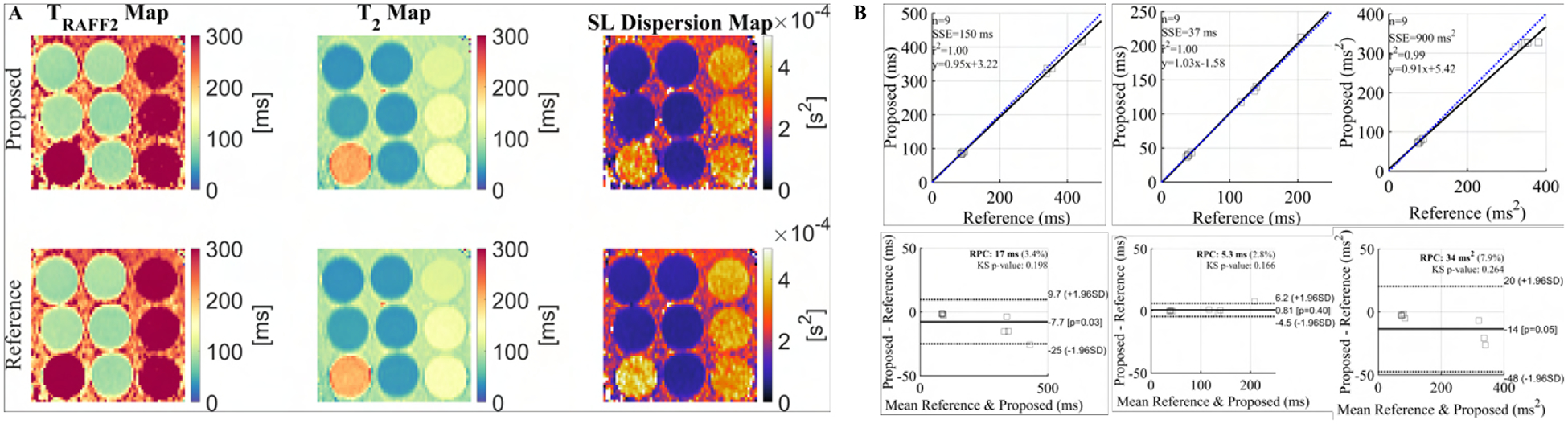
A) *T*_2_, *T*_*RAFF*2_, and spin-lock (SL) dispersion maps for the T1MES phantom using the proposed simultaneous sequence (top row) and the single-parameter reference maps (bottom row). B) Correlation and Bland-Altman plots for each parameter, showing an excellent correlation between the relaxation properties in phantom, in the expected in vivo range.

Joint *T*_*RAFF*2_ and *T*_2_ values obtained with the proposed sequence exhibited excellent correlation (*R*^2^ = 1.00) with individually acquired reference *T*_*RAFF*2_ and *T*_2_ values for the phantom vials (Fig. 2). A bias of −7.7*ms* was measured for *T*_*RAFF*2_, 0.8*ms* for *T*_2_, and −14*ms*^2^ for SL dispersion. The limits of agreement were ±17.4*ms* (*T*_*RAFF*2_), ±5.4*ms* (*T*_2_), and ±34*ms*^2^ (SL dispersion).

**Fig. 3.**
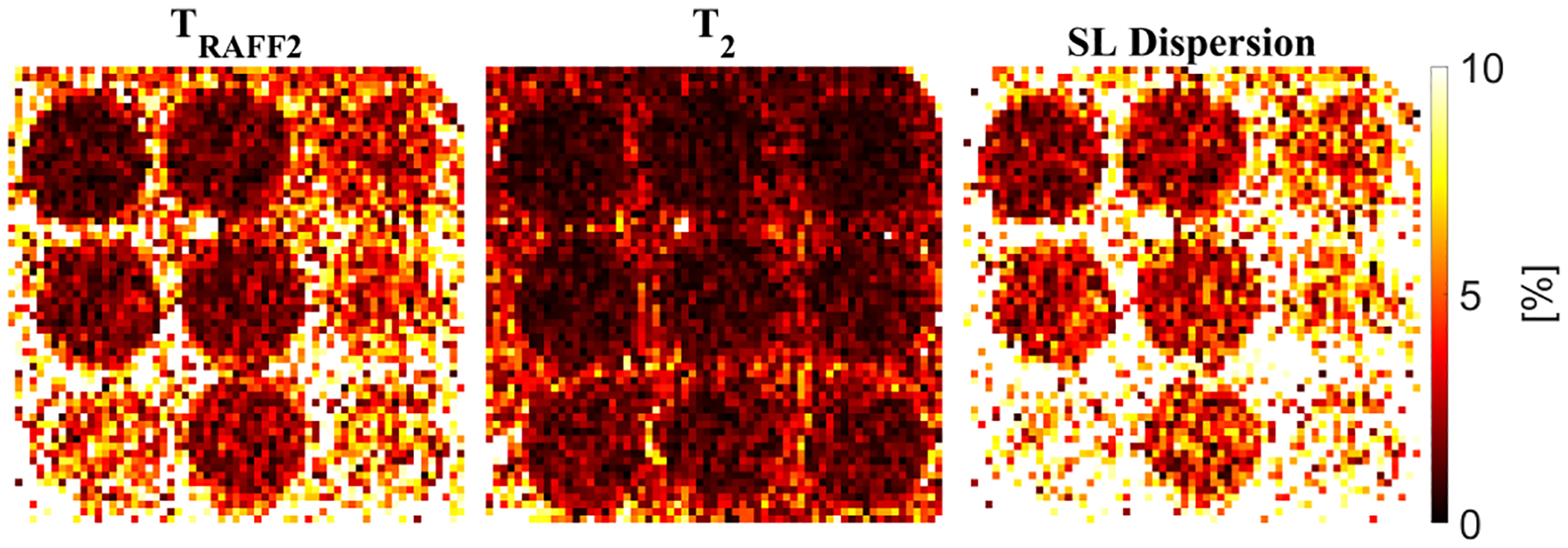
Coefficient of variation (CV) across three measurements using the proposed simultaneous *T*_*RAFF*2_ and *T*_2_ mapping sequence. All vials show a CV lower than 10% demonstrating excellent reproducibility of the proposed sequence.

SL dispersion maps yield good sensitivity to changes in the vials of the T1MES phantom (range 0.7 – 3.3 × 10^*−*4^*s*^2^). The three imaged biomarkers showed a coefficient of variation of less than 10% between repeated scans, indicating good short-term reproducibility (Fig. 3).

### B. In vivo

**Fig. 4.**
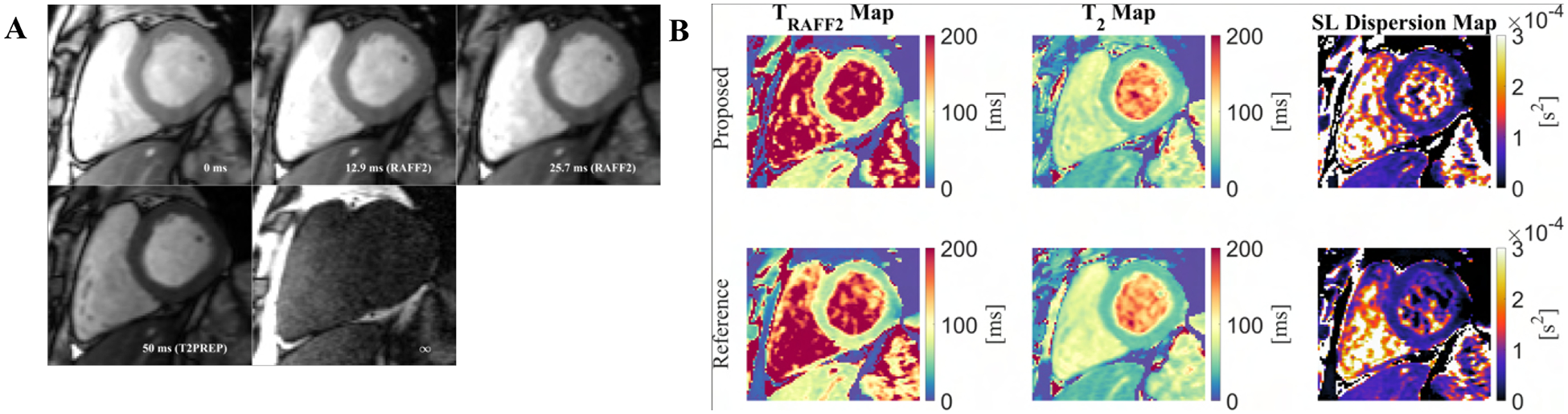
A) Baseline images for the proposed simultaneous *T*_*RAFF*2_ and *T*_2_ mapping sequence with the corresponding preparation times. B) In vivo myocardial *T*_*RAFF*2_, *T*_2_, and spin-lock (SL) dispersion maps were obtained with the proposed sequence and with the single-parameter sequences (Reference). Following image registration, the dispersion map is obtained by subtracting *T*_2_ from *T*_*RAFF*2_ and dividing by the RAFF2 pulse power. All maps present high visual image quality, homogeneous myocardial signal and no $B_{1}^{+}$ or *B*_0_ artifacts are visible.

In vivo *T*_*RAFF*2_ and *T*_2_ maps obtained with the proposed sequence achieved high visual map quality, and no $B_{1}^{+}$ or *B*_0_ related artifacts were visible (Fig. 4). The average (± std) myocardial *T*_*RAFF*2_ and *T*_2_ obtained with the proposed sequence (68.0 ± 10.7 and 44.0 ± 4.0 ms, respectively) were comparable to the reference methods (62.7 ± 11.7 and 41.2 ± 2.4 ms, respectively). The myocardial SL dispersion map resulted in an excellent depiction of the myocardium with a small increase in variability when compared to the SL dispersion obtained with the single-parameter reference maps (0.4 ± 0.2 and 0.3 ± 0.2 × 10^−4^ *s*^2^, respectively).

**Fig. 5.**
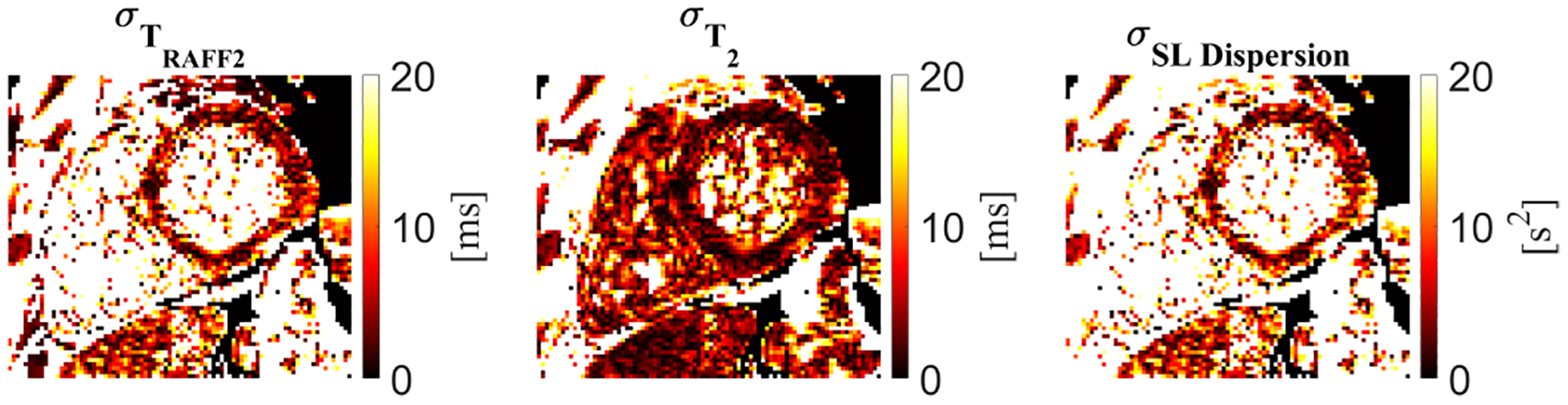
Standard deviation (*σ*) maps computed from the fit residuals to obtain a spatially resolved estimation of the *T*_*RAFF*2_, *T*_2_, and spin-lock (SL) dispersion precision.

Low variability was present for myocardial *T*_*RAFF*2_ (5.2 ± 5.1 ms), *T*_2_ (2.4 ± 2.1 ms), and SL dispersion (5.7 ± 5.5 *s*^2^) SD maps (Fig. 5). The homogeneous myocardial value in the SD maps indicates a high precision of the proposed sequence.

## IV. DISCUSSION

In this work, we propose a method for approximate SL dispersion mapping in the heart by measuring *T*_*RAFF*2_ and *T*_2_ in a single 16s BH at 3T. Homogeneous *T*_*RAFF*2_, *T*_2_, and SL dispersion maps of high visual quality were obtained for phantom and in vivo. Our preliminary phantom results showed good sensitivity for a wide range of expected myocardial values. In a healthy volunteer, a clear depiction of the myocardium with no visible $B_{1}^{+}$ or *B*_0_ artifacts was achieved.

The proposed sequence design includes a saturation-prepared image, in addition to the RAFF2- and *T*_2_-prepared images. This allows the use of a four-parameter fit model, which has previously been shown to capture the effects of the image readout and, therefore, enables a more reproducible mapping across different protocol parameters [10]. However, the model assumes a negligible asymptotic magnetization for the exponential decay functions. This assumption has previously been used for RAFF decay. However, experimental validation may be warranted to ascertain bias-free mapping with the proposed sequence.

Similar to conventional relaxation times, *T*_1*ρ*_ relaxation times are driven by several factors, including diffusion, chemical exchange, J-coupling, and spin-spin interaction. Alternatively, SL dispersion has been shown to provide a more specific measure of exchange effects by separating the relaxation rate, exchange rates, and frequency offsets of exchanging pools. Thus, SL dispersion may offer a valuable tool to increase specificity in the detection of pathological remodelling. Therefore, clinical use in ischemic and non-ischemic heart disease will be the subject of further investigation.

Previous works have demonstrated a good sensitivity of *T*_1*ρ*_ to fibrosis in the liver [13], however, limited studies have been performed for in vivo myocardial fibrosis, especially at high fields. This can be attributed to the high specific absorption rate (SAR) of the multiple *T*_1*ρ*_ RF pulse amplitude required in a conventional SL dispersion mapping. RAFF offers an attractive alternative to continuous wave SL due to lower SAR burden (30% reduction [7]) and better resilience to off-resonances. At the same time, RAFF may be more reflective of a single SL frequency component compared to adiabatic *T*_1*ρ*_. Since *T*_1*ρ*_ relaxation depends on the orientation of the magnetization and the effective field, as well as the effective field strength, *T*_1*ρ*_ relaxation is non-constant throughout adiabatic *T*_1*ρ*_ preparation pulses. In RAFF preparations, the effective field strength, as well as the fictitious field components, giving rise to the sweep, are kept constant and identical throughout the preparation. Thus, the proposed simultaneous *T*_*RAFF*2_ and *T*_2_ mapping sequence is an excellent candidate for the clinical application of SL dispersion rate due to the lower requirements in SAR while still approximating exchange rate information. The proposed approach is readily available for use in the clinical environment, and future work will include the evaluation of the proposed approximate SL dispersion method for the detection of myocardial infarction in a large cohort of patients.

## V. CONCLUSION

Our results show promising potential for mapping approximate myocardial spin-lock dispersion in a single BH using *T*_*RAFF*2_ mapping with clinically tolerable SAR. The proposed sequence obtains simultaneous *T*_*RAFF*2_ and *T*_2_ maps and yields high visual image quality, homogeneous signal, no $B_{1}^{+}$ or *B*_0_ artifacts, and excellent reproducibility. Thus, approximate spin-lock dispersion mapping with *T*_*RAFF*2_ bears great promise for clinical sensitivity to pathological remodeling and is an excellent candidate for the non-contrast assessment of scar with cardiac MRI.

## Clinical relevance—

*T*_*RAFF*2_ and *T*_2_ imaging promises non-contrast assessment of scar and focal fibrosis in a single breath-hold using approximate spin-lock dispersion mapping.
